# Navigating Uncertainty: The Future of Nepal Demographic and Health Survey and Nepal Health Facility Survey in Evolving Public Health Landscape in Nepal

**DOI:** 10.31729/jnma.8984

**Published:** 2025-06-30

**Authors:** Deepak Paudel

**Affiliations:** 1 Nepal Public Health Association, Lalitpur, Nepal; 2 Center for International Health, Ludwig Maximilians University, Munich, Germany

**Keywords:** *Demographic and Health Survey*, *Health Facility Survey*, *Nepal*, *USAID*

## Abstract

Effective data systems are essential for driving focus and processes within health systems. Demographic and Health Surveys (DHS) and Health Facility Surveys (HFS) play a critical role in providing nationally representative data on service quality, health indicators, and resource allocation. Although the next round of Nepal Health Facility Survey was scheduled for 2026 and Nepal Demographic and Health Survey for 2027, these surveys face uncertainty due to recent decisions by the new U.S. administration to cut foreign aid, including support for the DHS project. In Nepal, data from DHS and HFS are indispensable for evaluating health systems, shaping evidence- based policies, and monitoring public health progress. However, funding uncertainties threaten to create data gaps, may distort health sector priorities, and diminish opportunities for developing future leaders in health sector. This situation highlights the urgent need for innovative solutions, investments in robust routine data systems, and strengthened collaboration among stakeholders to ensure the continuity of vital health system data.

## INTRODUCTION

It is often said that "what gets measured, gets done," a principle that effectively underscores the importance of data in directing attention and driving key processes within systems.^1^ In health systems, diverse types of data are generated and employed to inform program decisions, allocate limited resources strategically, monitor progress over time, and compare the performance of various units. These systems generate extensive volumes of data, which not only serve as a foundation for patient care but also play a pivotal role in improving the availability, accessibility, utilization, and quality of healthcare services.^2^

Demographic and Health Surveys (DHS) are nationally representative household surveys conducted in over 85 countries since 1984.^3^ In Nepal, the survey began with the National Fertility, Family Planning, and Demographic Survey in 1991.^4^ Since 1996, the standard surveys have been conducted every five years, providing valuable coverage and health outcome indicators for health program planning, prioritization and evaluation.^5^

Similarly, Health Facility Surveys (HFS), also referred to as Service Provision Assessments, collect nationally representative data on service availability and quality of care measures within a country's health system.^6^ Conducted in over 30 countries, these surveys are often adapted to specific national needs and conducted at varying frequencies. They are particularly valuable for nations where routine data systems face challenges such as irregular reporting or issues with data quality. There were two rounds of HFSs in Nepal, in 2015 and 2021.

## IMPORTANCE OF DATA AND DATA QUALITY

High-quality, real-time data on disease burden and health sector performance are essential for informed decisionmaking and effective resource allocation.^7^ Health care managers rely on accurate data throughout every stage of the program cycle, including planning, implementation, and evaluation. Reliable data serves as the backbone of program planning, target setting, progress monitoring, and impact assessment. However, despite the availability of various datasets in a country, many suffer from significant challenges such as under-reporting, inconsistent reporting, and poor- quality data, which undermine their reliability and utility and demands for standardized survey data collected through robust tools and processes.

## KEY DATA SOURCES IN NEPAL'S HEALTH SECTOR

In Nepal's Health system, various data sources and systems are used to plan, execute and monitor health programs. Following are the key groups of data sources for program planning and monitoring used on a regular basis. (Table 1)

**Table 1:** Key Data Sources, Objectives, Frequency, and Supporting Partners

Source	Key Objectives	Frequency (survey years)	Key Supporting Partner
National Population and Housing Census	To develop a set of benchmark data for programs and to provide distribution of population by demographic, social and economic characteristics	Every 10 years, Since 1911, latest in 2021	UNFPA
Civil Registration and Vital Statistics	To provide vital statistics for policy and monitoring purposes, and aligning, including legal identity for citizens	Since 1997, Regular through the office of local registrar	
Nepal Demographic and Health Survey	To present up-to-date estimates of basic demographic and health indicators	Every 5 years, (1996, 2001, 2006, 2011, 2016, 2022)	USAID
Nepal Health Facility Survey	To provide a comprehensive picture of the strengths and weaknesses of the service delivery environment by type of services and delivery units (both public and private)	Every 5 years (2015, 2021)	USAID
Nepal Multiple Indicator Cluster Survey	To assess situation of children and women	Every 5 years (1995-97, 2010, 2014, 2019, 2025-25)	UNICEF
Nepal Living Standards Surveys	To understand household welfare, to monitor the key economic and governance reforms on poverty reduction and well-being	Every 7 years (1995-96, 2003-04, 2010-11, 2022-23)	World Bank
Health Management Information System	To collect, store, process and report health service delivery statistics to assist monitoring, evaluation and policymaking	Regular, data accessible for health system officials on regular basis, Consolidated data on annual basis for public.	
Data projection and modelling by various organizations e.g. World Bank, WHO, UNICEF, Institute of Health Metrics and Evaluation	To provide internationally comparable data on morbidity, mortality, disease burden and other socio-economic variables for different time points (especially when the data from routine system or other reliable surveys are not available)	Regularly updated and shared in their portal	Respective organization

## IMPORTANCE OF HEALTH SURVEY DATA

Data for evaluating health system and impact at population level indicators are generated by two major global survey programs: DHS and HFS. These surveys are primarily funded by USAID, and implemented in leadership and close coordination with host governments.

The Nepal DHS survey provides invaluable information to program planners, academics, and researchers. The data is publicly available, free to use, and well-organized datasets offer further analysis to generate insights into trends over time and across countries as well as to evaluate large- scale public health programs. Data collection is conducted routinely using a uniform methodology, with standardized core survey modules ensuring comparability across time and locations.^8^ As these surveys primarily focus on maternal, newborn, child health, and family planning, they do not capture comprehensive information on all health issues such as non-communicable diseases or infectious diseases e.g. tuberculosis and malaria. Moreover, the data is largely based on verbal reports from women and household heads.

In addition to household surveys, the Nepal HFS, a locally adapted version of the internationally standardized Service Provision Assessment, is a critical tool for measuring health system performance. It focuses on assessing the availability of basic and essential health services, health facility readiness to deliver quality care, adherence to accepted standards for service provision, and tracking health system performance at federal and provincial levels. For instance, the NHFS 2015 and 2021 were instrumental in evaluating the status of basic health service delivery, assessing health system performance during the Nepal Health Sector Strategy 2015-2020, and identifying process barriers to delivering quality healthcare services in both public and private facilities.

## CURRENT SITUATION OF NHFS 2026 AND NDHS 2027

Maintaining the 5-year cycle of the Nepal Demographic Health Survey (NDHS) and Nepal Health Facility Survey (NHFS), the next NHFS is scheduled for 2026 and the next NDHS is scheduled for 2027. Historically, the United States Agency for International Development (USAID) has been the key donor supporting Nepal's Ministry of Health and Population in funding these surveys and providing essential technical assistance for their planning and implementation. The survey design, implementation and reporting require rigorous process and mobilization of huge number of trained and supervised enumerators. However, recent decisions by the new administration in the United States have led to the termination of many USAID projects, including the Demographic and Health Survey.^9^ Consequently, preparations for the upcoming rounds of NHFS and NDHS is disrupted, raising significant concerns about the availability of data for monitoring health system performance and health outcomes in 2026 and beyond.

Data from these surveys are used in many national level documents, including in monitoring of Sustainable Development Goals, National Periodic Plan, and National Health Sector Strategic Plan. For example, a number indicators in the results framework of National Health Sector Strategic Plan 2023-203010 rely on these surveys:

5 goal level indicators of the strategy rely on NDHS as a data source (i.e. maternal mortality, neonatal mortality, under-five mortality, % stunting, and total fertility rate)

5 output level indicators of the strategy rely on NHFS as a data source (i.e. % sanction post filled, % health facilities with tracer amenities, % clients satisfied with tracer services, % health facilities meeting tracer standards for quality of care, % health facilities with capacity to provide selected laboratory services as per standard)

3 output level indicators of the strategy rely on NDHS as a data source (i.e. adolescent birth rate, % household within 30-minutes of travel to the health facility, prevalence of wasting)

In addition to these indicators of the Strategic Plan, data from NDHS and NHFS are integral to other health-related federal and provincial level strategic plans and roadmaps e.g. National Safe Motherhood and Newborn Health Roadmap. In addition to direct use of data from these surveys, global organizations use the data to track progress and model projections during periods when data is unavailable e.g. to estimate maternal mortality ratio by World Health Organization (WHO). If the upcoming cycles of NDHS and NHFS are delayed or canceled, it would create a critical gap in the data point. It will also likely to distort health sector priorities due to this data void and severely hindering efforts to monitor progress. This disruption would represent a significant setback for public health and program monitoring professionals.

Moreover, the data from NDHS and NHFS is traditionally analyzed beyond the standard survey reports to uncover issues and propose solutions for various public health challenges. A delay or cancellation of these surveys would diminish the ability to address these issues effectively, compounding the challenges faced by the healthcare system. The decision also affects training of health professionals, researchers, and scientists as they will face two major setbacks: the discontinuation of courses on survey methodologies and the loss of access to survey data, which is widely used by undergraduate, master's, and doctoral students for their academic research, particularly in low- and middle-income countries. These challenges will critically undermine the development and preparation of future leaders in national and global health.^11^

## WHAT ARE THE OPTIONS?

As the timeline for these surveys' approaches, uncertainty regarding their executing due to changes in USAID funding scenario calls for immediate attention from national program leaders and managers to explore alternative solutions. This situation underscores the urgent need for further investment in routine information systems to collect, compile, and report reliable data, especially by strengthening the Health Management Information System and Civil Registration and Vital Statistics systems.

Given the importance and reliability of data provided by these surveys, the government and development partners should investigate the feasibility of conducting them using domestic revenue and financial support from other stakeholders. Joint consultations are required to determine the critical elements of the survey, cost-efficient methodology, and opportunities for cost reduction through the use of in-country expertise and resources. Additionally, alignment of timeline and content with other large-scale surveys (e.g., UNICEF-supported Multiple Indicator Cluster Survey) or smaller-scale efforts (e.g., WHO-supported STEPwise approach to NCD risk factor surveillance Survey) should be explored to ensure efficiency and harmonization.

Furthermore, this is an opportune time to evaluate the appropriateness and feasibility of establishing a Health and Demographic Surveillance System as an alternative to these surveys. Such a system would begin with an initial census of each household within a demographic surveillance area, followed by annual visits to collect and update detailed household data.^12^ Lessons learned from some pilots in various locations within Nepal, e.g. in Bhaktapur,^13^ longitudinal data from Chitwan Valley Family Study^14^ and in Gandaki and Madhesh Provinces as the Family Health Profile initiative should be carefully evaluated and considered.

It is imperative for the Ministry of Health, National Planning Commission, and National Statistics Office to collaboratively assess the potential impact of changes in USAID foreign aid on these surveys and national data systems, identify critical needs, and outline feasible options. This collaborative effort should then extend to engaging development partners, researchers, and academia to devise the best possible solutions that balance national priorities with available resources.
